# Stigma toward people with COVID-19 among Bangladeshi older adults

**DOI:** 10.3389/fpubh.2022.982095

**Published:** 2022-09-13

**Authors:** Sabuj Kanti Mistry, A. R. M. Mehrab Ali, Uday Narayan Yadav, Md. Nazmul Huda, Md. Mahmudur Rahman, Manika Saha, Md. Ashfikur Rahman, David Lim, Saruna Ghimire

**Affiliations:** ^1^Department of Health Research, ARCED Foundation, Dhaka, Bangladesh; ^2^Centre for Primary Health Care and Equity, University of New South Wales, Sydney, NSW, Australia; ^3^BRAC James P. Grant School of Public Health, BRAC University, Dhaka, Bangladesh; ^4^Department of Public Health, Daffodil International University, Dhaka, Bangladesh; ^5^National Centre for Epidemiology and Population Health, Research School of Population Health, The Australian National University, Canberra, ACT, Australia; ^6^Translational Health Research Institute, School of Medicine, Western Sydney University, Campbeltown, NSW, Australia; ^7^Research, Monitoring and Information Management Organization/Institutions, Deep Eye Care Foundation, Rangpur, Bangladesh; ^8^Department of Human-Centred Computing, Faculty of Information Technology, Monash University, Clayton, VIC, Australia; ^9^Development Studies Discipline, Khulna University, Khulna, Bangladesh; ^10^School of Health Sciences, Western Sydney University, Campbeltown, NSW, Australia; ^11^Department of Sociology and Gerontology and Scripps Gerontology Center, Miami University, Oxford, OH, United States

**Keywords:** stigma, COVID-19, older adults, aged, Bangladesh

## Abstract

The onset of the coronavirus disease (COVID-19) pandemic and its overwhelming physical and mental health burden can result in stigmatization toward the disease and those affected. This study aimed to measure the prevalence of COVID-19-related stigma and its associated factors among older people in Bangladesh. This cross-sectional study was conducted among 1,045 Bangladeshi older adults aged 60 years and above through telephone interviews in September 2021. The outcome was measured using an eight-point Stigma Scale, adapted to the Bengali language. Level of stigma was indicated by the cumulative score of the eight-items, ranging from 0 to 8, with a higher score indicating a higher level of stigma. On average, participants had stigmas on three of the eight items, and 62.6% had a high stigma score. The most prevalent stigmas were as follows: COVID-19 is a punishment from God (79.3%), patients with previous COVID-19 must be isolated (67.3%), and people infected with COVID-19 did not meet hygiene standards (63.9%). Participants who lived in rural areas (β: 0.67, 95% CI: 0.39 to 0.95) and who perceived needing additional care during the pandemic (β: 0.35, 95% CI: 0.09 to 0.60) had a higher average stigma score, whereas stigma scores were lower among unemployed/retired participants (β: −0.22, 95% CI: −0.45 to 0.00). The study findings suggest implementing interventions to raise awareness through appropriate health literacy interventions and mass media campaigns.

## Introduction

The novel coronavirus disease (COVID-19) pandemic is recognized as one of the biggest global public health challenges of the century. According to the Director-General of the World Health Organization, “Our greatest enemy right now is not the virus itself. It's fear, rumors, and stigma” (1). Stigma is an important public health issue because of its direct correlation with adverse physical and mental health (2). Furthermore, stigma creates constraints to access to health and social services, disrupts health-seeking behavior, and creates social discrimination, exclusion, mental distress, and violence (3). Stigma may lead to distress and can affect early detection and timely treatment (4). Delayed diagnosis is linked with prognostic deterioration of the disease among vulnerable groups such as the older population, facilitating the quick spread of the infection, and increasing disease severity and complications (5). Stigma is also closely related to discrimination and injustice (6).

Despite its strong relationship with adverse health consequences, historically, stigma has been prevalent during the peak of many infectious diseases, including HIV/AIDS, tuberculosis and COVID-19 (7). Several factors facilitate stigma, including the lack of awareness, misinformation, fake news, extreme fears, and anxiety about the diseases, which are widespread during the COVID-19 pandemic (8). Misconceptions about various aspects of the pandemics as well as fear of the disease further reinforce the stigma in societies (9, 10). In line with these, evidence suggests that facilitators of stigma are widespread. Studies from Jordan (11), Uganda (12), and Lebanon (13) found COVID-19-related stigma to be prevalent among the general population. Notably, these studies include younger adults, and there is scarce COVID-19-related stigma research among the older population. In a study from Ghana, patients with COVID-19 reported experiencing various forms of stigma, e.g., stereotyping, social exclusion, mockery, finger-pointing, and insults (14). In another study from Ghana, nearly half of the participants exhibited stigma and discriminatory tendencies toward COVID-19 survivors (15). In the study from Malaysia, COVID-19-recovered participants expressed experiencing being labeled and blamed by the people around them (16). For several reasons, mentioned in the following paragraph, we believe that the level of COVID-19 stigma would be higher among older adults, in general, and more especially among Bangladeshi older adults, which warrants a separate study. In resource-constraint countries, such as Bangladesh, some critical social factors, including poverty, poor living conditions, low illiteracy rates, ethnic disparity, age, and gender disparities, can make COVID-19-related stigma conditions more complicated than in other resource-rich countries (4, 17). In line with the international studies, stigma against specific groups, such as healthcare professional, returning migrant workers, and persons with a travel history from COVID-19 hotspot countries (18–21), was also observed in Bangladesh (22–24).

The population groups most vulnerable to stigma are those directly and indirectly affected by the disease or those highly susceptible to the infections (6). Evidence has already proved that older adults (the age group of 65 and above) are one of the most vulnerable groups impacted by the COVID-19 disease globally. Compared to other age groups, older adults are at increased risk of developing severe disease, requiring hospitalization, and dying from COVID-19. According to Kaiser Family Foundation (KFF) data, as of 19 September 2021, about 80% of COVID-19 deaths were among those aged 65 and above (25). Recent studies conducted among older Bangladeshi adults aged 60 years and above revealed significant misconceptions and an overwhelming fear of COVID-19 (9, 10). Bangladeshi older adults also faced difficulty in getting medicine and receiving COVID-19-related information during this pandemic (26). The literacy rate and access to health information, two important determinants of stigma, are low among Bangladeshi older adults (27). While all these characteristics suggest that stigmas can be higher among Bangladeshi older adults, there is no evidence from Bangladesh regarding the extent to which COVID-19-related stigmas are present among them. In this context, using an analytical approach, our paper investigates the level of stigma toward people with COVID-19 and its associated factors among older adults in Bangladesh.

## Materials and methods

### Study design and participants

This cross-sectional study was conducted remotely through telephone interviews in September 2021 by the Aureolin Research, Consultancy and Expertise Development (ARCED) Foundation. The primary challenge for this study was to develop a sampling frame to select the participants during the COVID-19 pandemic, and thus, we utilized a pre-established registry, developed through merging the contact information of households from 10 different community-based studies accomplished by Aureolin Research, Consultancy, and Expertise Development (ARCED) Foundation during 2016–2020, which included households from all eight administrative divisions of Bangladesh, as a sampling frame. Based on the population distribution of older adults by geography in Bangladesh, we adopted a probability proportionate to size (of the eight-division) approach to select older adults in each division (28). The inclusion criterion was the minimum age of 60 years. Considering 50% prevalence with a 5% margin of error, at the 95% level of confidence, 90% power of the test, and 95% response rate, a sample size of 1,096 was required. However, of the 1,096 eligible participants approached, 1,045 responded to the study with an overall response rate of approximately 95%.

### Measures

#### Outcome measure

Level of stigma was measured using the eight-item Stigma Scale, which was previously translated and validated in the Bengali language (11). Each item was nominally coded as a yes/no statement where a correct response scored 0 and each stigma scored 1. The cumulative of the eight items generated a stigma score for each participant. The cumulative score of the eight-items ranged from 0 to 8, with a higher score indicating a higher level of stigma. We further classified COVID-19-related stigma into low (if the stigma score was below the mean of the scale value, i.e., <4) or high (if the stigma score was equal to or higher than the mean of the scale value, i.e., ≥4). The scale is reliable among the study participants, as indicated by the high internal consistency (Cronbach's alpha 0.74).

#### Explanatory variables

Explanatory variables considered in this study were age (categorized as 60–69 and ≥70), sex (male/female), marital status (married/without partner), formal education (no/yes), family size ( ≤ 4 or >4), family income in Bangladeshi Taka (BDT) (<5,000, 5,000–10,000, >10,000), residence (urban/rural), current work arrangements (employed and unemployed/retired), living arrangements (living alone or with family), walking distance to the nearest health center (<30 /≥30 min), memory or concentration problems (no problem/low memory or concentration), the presence of prevalent non-communicable chronic conditions (yes/no), concerned about COVID-19 (not concerned/somewhat to very concern), overwhelmed by COVID-19 (hardly, sometimes/often), perceived to be at risk of COVID-19 (low risk/high), difficulty in obtaining food, medicine, routine medical care, and earning during COVID-19 (no/yes), perceived isolation (hardly, sometimes/often), frequency of communication with friends and family during COVID-19 (less than previous/same as previous), perceived that family members are non-responsive (yes/no), and that they required additional care during COVID-19 (yes/no). Self-reported information on pre-existing medical conditions, such as arthritis, hypertension, heart diseases, stroke, hypercholesterolemia, diabetes, chronic respiratory diseases, chronic kidney disease, and cancer, was collected.

### Data collection tools and techniques

A pre-tested semi-structured questionnaire was used to collect the information through a telephone interview. Data collection was accomplished electronically using SurveyCTO mobile app (https://www.surveycto.com/) by trained research assistants, who were recruited based on previous experience administering health surveys using the same electronic platform. The research assistants were trained extensively before the data collection through Zoom meetings.

The English version of the questionnaire was first translated into Bengali language and then back-translated to English by two researchers to ensure the contents' consistency. The questionnaire was then piloted among a small sample (*n* = 10) of older adults to refine the language in the final version. The tool used in the pilot study did not receive any corrections/suggestions from the participants in relation to the contents developed in the Bengali language.

### Statistical analysis

The distribution of the variables was assessed through descriptive analysis. We used linear regression models to explore the factors associated with stigma. The initial model was run with all potential covariates, and then, using the backward elimination criteria with the Akaike information criterion (AIC), the variables for the final model were selected and executed. Adjusted beta-coefficient and associated 95% confidence interval (95% CI) were reported. We also performed the model diagnostics, such as normality of the residuals and multicollinearity using variance inflation factor (VIF). All analyses were performed using the statistical software package Stata (version 14.0).

## Results

### Characteristics of the participants

Table 1 describes the sociodemographic and lifestyle characteristics of participants. A higher proportion of participants were aged 60–69 years (75.6%), male (59.3%), married (76.5%), without formal schooling (51.7%), from a family with more than four members (66.8%), with monthly family income between 5,000 and 10,000 BDT (44.9%), from rural areas (82.6%), unemployed/retired (61.1%), living with family members (94.9%), and residing <30 min of walking distance to the nearest health center (55.6%). Many participants also reported pre-existing non-communicable chronic conditions (57.2%). The majority of participants reported that they were concerned about (66.7%) and overwhelmed by (67.9%) COVID-19 but perceived themselves as at low risk of developing COVID-19 (72.7%). In total, 37.2% of the participants reported reduced communication during the pandemic, and many reported experiencing difficulties in obtaining food (50.3%) and earning money (72.3%). More than a quarter of the participants reported feeling isolated (31.3%) and perceived that they required additional care during the pandemic (26.3%), but their family members were non-responsive (29.4%).

**Table 1:** Characteristics of the participants (*N* = 1,045).

**Characteristics**	***n***	**%**
Administrative division		
Barishal	146	14
Chattogram	98	9.4
Dhaka	172	16.5
Mymensingh	69	6.6
Khulna	198	19
Rajshahi	145	13.9
Rangpur	161	15.4
Sylhet	56	5.4
Age (year)		
60–69	790	75.6
≥70	255	24.4
Sex		
Male	620	59.3
Female	425	40.7
Marital status		
Married	799	76.5
Without partner^a^	246	23.5
Formal schooling		
No	540	51.7
Yes	505	48.3
Family size		
≤ 4	347	33.2
>4	698	66.8
Family monthly income (BDT)^b^		
<5,000	121	11.6
5,000–10,000	469	44.9
>10,000	455	43.5
Residence		
Urban	182	17.4
Rural	863	82.6
Current work arrangements		
Employed	407	39
Unemployed/retired	638	61.1
Living arrangement		
Living with family	992	94.9
Living alone	53	5.1
Walking distance to the nearest health center		
<30 min	581	55.6
≥30 min	464	44.4
Problem in memory or concentration		
No problem	676	64.7
Low memory or concentration	369	35.3
Prevalent non-communicable chronic conditions		
No	447	42.8
Yes	598	57.2
Feeling concerned about COVID-19		
Not concerned	348	33.3
Somewhat to very concern	697	66.7
Feeling overwhelmed by COVID-19		
Hardly	334	32.1
Sometimes/Often	706	67.9
Self-perceived risk of COVID-19		
Low risk	760	72.7
High	285	27.3
Difficulty getting food during COVID-19		
No difficulty	514	49.7
Some difficulty	521	50.3
Difficulty getting medicine during COVID-19		
No difficulty	764	74.8
Some difficulty	258	25.2
Difficulty earning during COVID-19		
No difficulty	274	27.7
Some difficulty	714	72.3
Difficulty receiving routine medical care during COVID-19		
No difficulty	709	71
Somewhat difficulty	290	29
Frequency of communication during COVID-19		
Same as previous	656	62.8
Less than previous	389	37.2
Perceived isolation during COVID-19		
Hardly	718	68.7
Sometimes/Often	327	31.3
Perceived family members to be non-responsive		
No	738	70.6
Yes	307	29.4
Feeling that they required additional care during the pandemic		
No	770	73.7
Yes	275	26.3

^a^Includes widowed, separated, and never married.

^b^BDT ~0.012 USD.

### Prevalence of stigma

Table 2 shows the prevalence of stigma related to COVID-19 among the participants. On average, participants had stigma on three items (mean stigma score = 2.97 and range 0–8). We found that 62.6% of the participants had a high level of stigma. The most prevalent stigmas were as follows: infection with COVID-19 is a punishment from God (79.3%), previous patients with COVID-19 have to be isolated (67.3%), people who were infected with COVID-19 did not meet the standards for hygiene (63.9%), it is unsafe to deal with people who have been infected with COVID-19 (62.4%), and people who have been infected with COVID-19 should expect some restrictions on their freedom (59.6%). Other less prevalent yet notable stigmas related to COVID-19 included: it should not be allowed to work for those who have been infected with COVID-19 (33.8%), to be friends with someone who has been infected with COVID-19 (27.3%), and those infected with COVID-19 should be ashamed of themselves (18.4%).

**Table 2:** Prevalence of stigma among the participants (*N* = 1,045).

**Items of Stigma Scale**	***n***	**%**
Infection with COVID-19 is a punishment from God (yes)	829	79.3
Previous COVID-19 patients have to be isolated (yes)	703	67.3
People infected with COVID-19 did not meet the standards for hygiene (yes)	668	63.9
It is unsafe to deal with people who have been infected with COVID-19 (yes)	652	62.4
People who have been infected with COVID-19 should expect some restrictions on their freedom (yes)	623	59.6
It should not be allowed to work for those who have been infected with COVID-19 (yes)	353	33.8
You do not want to be a friend of someone who has been infected with COVID-19 (yes)	285	27.3
Those infected with COVID-19 should be ashamed of themselves?	192	18.4

### Factors associated with stigma

An initial regression model was run with all the variables presented in Table 1, and a final model was executed with variables retained from the initial model based on the lowest AIC values. The result of the final model is presented in Table 3. The Q-Q plot of the residuals of the model shows that the data are normally distributed, whereas VIF values of <10 for each variable (Supplementary Tables S1, S2) suggest the absence of multicollinearity. In both adjusted and unadjusted analyses, residence and perception that they need additional care during the pandemic was associated with higher stigma score. In the adjusted analysis, participants who lived in rural areas had 0.67 units (β: 0.67, 95% CI: 0.39 to 0.95) higher stigma than those living in urban areas. Likewise, participants who felt that they required additional care during the pandemic had 0.35 unit higher stigma score (β: 0.35, 95% CI: 0.09 to 0.60) than those who did not feel so. On the other hand, stigma scores were 078 unit lower among unemployed/retired participants than those employed (β: −0.22, 95% CI: −0.45 to 0.00).

**Table 3:** Factors associated with stigma among the participants (*N* = 1,045).

**Characteristics**	**Unadjusted**			**Adjusted**		
	**β**	***P***	**95% CI**	**β**	***P***	**95% CI**
Residence						
Urban	*Ref*			*Ref*		
Rural	0.68	<0.001	0.41, 0.96	0.67	<0.001	0.39, 0.95
Current occupation						
Employed	*Ref*			*Ref*		
Unemployed/retired	−0.21	0.072	−0.43, 0.02	−0.22	0.048	−0.45, −0.002
Frequency of communication during COVID-19
Same as previous	*Ref*			*Ref*		
Less than previous	0.11	0.321	−0.11, 0.34	0.18	0.137	−0.06, 0.41
Perceived isolation during COVID-19
Hardly	*Ref*			*Ref*		
Sometimes/often	0.21	0.071	−0.02, 0.44	0.15	0.223	−0.09, 0.38
Feeling that they required additional care during the pandemic
No	*Ref*			*Ref*		
Yes	0.36	0.005	0.02, 0.60	0.35	0.008	0.09, 0.60

## Discussion

This study assessed the COVID-19-related stigma and its associated factors among Bangladeshi older people. Overall, our study found a high prevalence of stigma among participants. Although we did not find any studies exploring COVID-19-related stigma among older adults in Bangladesh and worldwide, our findings are consistent with similar literature among younger people. Our findings are similar to studies among COVID-19 survivors (24–59 years) in Uganda (12), COVID-19-infected Jordanian people (18–65 years) (11), Lebanese adults (>18 years) (13), and Chinese adults (aged ≥18) living in the United States (8). These studies reported highly prevalent COVID-19-related stigma, ranging from 62 to 65%. Likewise, in two studies from Ghana, patients with COVID-19 reported experiencing stigma and discrimination (14, 15). In the study from Malaysia, COVID-19-recovered participants expressed experiencing being labeled and blamed by the people around them (16). While being novel among the older population, our study extends and supplements previous research among younger adults (8, 11–13) to enrich our understanding of COVID-19 stigma. Our study's finding may not be surprising and could be attributable to various factors. One potential reason for a high COVID-19-related stigma in older people in our study can be their limited literacy and inadequate understanding of the disease and how the SARS-CoV-2 virus is transmitted (11). Specifically, in Bangladesh, literacy rate is only 39.4% among older adults aged 65 years or above (29). Furthermore, given that the second wave of the COVID-19 pandemic was ongoing in Bangladesh during the data collection period, and COVID-19-related misinformation, fear, and panic may be other reasons for the observed stigma (30). As COVID-19-related mortality instigates fear and panic among individuals (8, 31), having family members, relatives, friends, colleagues, and neighbors die from the SARS-CoV-2 virus may be another reason for increasing COVID-19-related stigma (30). Irrespective of the reasons, prevalent stigma during the pandemic could threaten public health preventive measures. High COVID-19-related stigma may increase the risk of spreading the SARS-CoV-2 virus by delaying the early testing and detection of COVID-19, hampering individuals' health-seeking behaviors (32), and utilization of healthcare services (30). Furthermore, COVID-19-related stigma may increase individuals' psychological distress (12). Our findings suggest that it is vital to tailor interventions to this specific group of older people to reduce COVID-19-related prejudices and stigmas.

In our study, compared to urban residents, those living in rural areas in Bangladesh had a higher COVID-19-related stigma. Given that stigma is highly correlated with literacy and can be reduced with appropriate educational interventions (33), the wide gap in literacy rate between urban and rural Bangladesh (34) may explain this finding. Rural Bangladesh has a low literacy rate and limited access to health information (34). Studies have already shown that people with limited knowledge or education are more stigmatized than others (35). As such individuals with limited knowledge about COVID-19 transmission and prevention may have increased stereotypes and stigma related to the disease (36), additionally, they have limited awareness about the pandemic and increased fear of SARS-CoV-2 virus infection, which may further increase the likelihood of COVID-19-related stigma (37). Our finding highlighted the importance of providing older residents in rural areas of Bangladesh with appropriate knowledge about the prevention and transmission of COVID-19, which can potentially reduce COVID-19-related stigma among in the community.

In our study, stigma scores were also higher in the older adults who felt that they required additional care during the COVID-19 pandemic. No previously documented studies are available for cross-comparisons. The potential explanations for the finding could be attributed to personal insecurities amidst the pandemic. When a person is highly concerned about their inadequate medical support and care during COVID-19 and its consequence (e.g., possible deterioration of existing comorbidities, illness, death, etc.), mental trauma is likely to be high (38). Further, in the context of Bangladesh, where financial support from governmental and non-governmental organizations are limited (39) and financial and food insecurities are high, the COVID-19 pandemic has negatively impacted livelihood directly or indirectly (39). This disruption in household food supply and livelihood strategies may have exacerbated their fear and mental trauma (40). In addition, health facilities were disrupted, and routine medical care and medications were inaccessible in Bangladesh during the pandemic (26). Such concerns may have produced negative thoughts about COVID-19, leading to COVID-19-related stigma (30).

We also found that unemployed/retired participants had lower COVID-19-related stigma than those employed. We did not find any study in the literature to compare this finding with. The probable reason is that unemployed/retired older people mostly stay at home and get adequate time to read newspapers, watch live television news, and engage in interactions and communications with families, which can be a vital source of COVID-19 information (10). Furthermore, they are less fearful about the infection of COVID-19, given that their limited exposure at the workplace or outside of the home. Thus, they may be less fearful and less stigmatized against COVID-19 (30).

### Strengths and limitations of the study

To the best of our knowledge, this is the first study that explored the level of COVID-19-related stigma and associated factors among older people in Bangladesh. However, the study has specific limitations. First, our research was cross-sectional in nature. Therefore, causality cannot be established. Second, our study is limited to quantitative analysis, as we did not explore the qualitative aspects of older people's COVID-19-related stigma.

## Conclusion

Overall, we found that the prevalence of stigmas related to COVID-19 is high among the older population in Bangladesh, which has an implication for policy and practice in COVID-19 management and moving beyond the WHO's world goal of 70% fully vaccinated by mid-2022. It is important that policymakers and public health practitioners working in this space are cognizant of the prevalent stigma while designing and planning for mass media campaigns to inform, educate, and counter the stigma associated with COVID-19. Failure to consider the context for stigma may result in suboptimal reach and social exclusion of the people affected with COVID-19 in Bangladesh. Given the high level of COVID-19 stigma in rural areas of Bangladesh, initiatives should be directed toward this targeted population.

## Data availability statement

The original contributions presented in the study are included in the article/Supplementary material, further inquiries can be directed to the corresponding author/s.

## Ethics statement

The Institutional Review Board of the Institute of Health Economics, University of Dhaka, Bangladesh approved the study protocol (Ref: IHE/2020/1037). All participants enrolled in the study provided voluntary verbal informed consents.

## Author contributions

SKM conceived and designed the study. MMR and SKM carried out the data analysis and interpretation of the result. SKM, AMA, UNY, MNH, MMR, MS, MAR, and DL contributed to the first draft of the manuscript. SG, UNY, DL, and MNH extensively edited the first draft to finalize. All authors approved the final version of the manuscript.

## Conflict of interest

The authors declare that the research was conducted in the absence of any commercial or financial relationships that could be construed as a potential conflict of interest.

## Publisher's note

All claims expressed in this article are solely those of the authors and do not necessarily represent those of their affiliated organizations, or those of the publisher, the editors and the reviewers. Any product that may be evaluated in this article, or claim that may be made by its manufacturer, is not guaranteed or endorsed by the publisher.
