# Supplementary material for: Stigma toward people with COVID-19 among Bangladeshi older adults
Source: Front Public Health. 2022 Sep 13;10:982095. doi: 10.3389/fpubh.2022.982095 (PMC9514800; doi:10.3389/fpubh.2022.982095)
Supplement: Supplementary file 1 [file Table_1.DOCX]

| SM 1: Multicollinearity diagnosis result | | |
| --- | --- | --- |
| Characteristics | **VIF** | **1/VIF** |
| Residence | 5.75 | 0.17 |
| Current occupation | 2.58 | 0.39 |
| Frequency of communication during COVID-19 | 1.62 | 0.62 |
| Feeling isolated from others | 1.51 | 0.66 |
| Feeling that they required additional care during the pandemic | 1.42 | 0.70 |
