# Supplementary material for: Stigma toward people with COVID-19 among Bangladeshi older adults
Source: Front Public Health. 2022 Sep 13;10:982095. doi: 10.3389/fpubh.2022.982095 (PMC9514800; doi:10.3389/fpubh.2022.982095)
Supplement: Supplementary file 2 [file Data_Sheet_1.docx]

**SM 2: Normality q-q plot**
